# Preparation of NiAl_2_O_4_-Based Flexible Substrates for Metamaterials with Negative Dielectric Properties

**DOI:** 10.1038/s41598-018-33295-0

**Published:** 2018-10-08

**Authors:** Md Atiqur Rahman, Eistiak Ahamed, Mohammad Rashed Iqbal Faruque, Mohammad Tariqul Islam

**Affiliations:** 1 Space Science Center (ANGKASA), Universiti Kebangsaan Malaysia, 43600 UKM Bangi, Selangor Malaysia; 2 Centre of Advanced Electronic & Communication Engineering, Universiti Kebangsaan Malaysia, 43600 UKM Bangi, Selangor Malaysia

## Abstract

Various techniques are commonly used to produce nano-crystalline NiAl_2_O_4_ materials; however, their practical applications in the microwave region remain very limited. In this work, flexible substrates for metamaterials containing two different concentrations of NiAl_2_O_4_ (labelled Ni_36_ and Ni_42_) have been synthesised using a sol-gel method. The formation of spinel structures in the synthesised materials is confirmed, and their crystalline sizes are determined using scanning electron microscopy, X-ray diffraction, and energy dispersive X-ray techniques. The dielectric properties, conductivities, loss tangents, and other parameters of the NiAl_2_O_4_-based substrates are analysed to evaluate their applicability as dielectric materials for the microwave frequency range. The obtained results show that the fabricated Ni_36_ and Ni_42_ nickel aluminates possess dielectric constants of 4.94 and 4.97 and loss tangents of 0.01 and 0.007, respectively; in addition, they exhibit high flexibility and light weight, which make them suitable for applications as metamaterial substrates. The synthesised structures are also validated experimentally using a commercially available electromagnetic simulator; as a result, double negative behaviour of the flexible metamaterials has been observed. Furthermore, it is found that the prepared NiAl_2_O_4_ substrates can be used in the S-, C-, and X-bands of the microwave frequency region.

## Introduction

Metamaterial is a macroscopic engineered composite of periodic structure with unusual electromagnetic properties that is unobtainable from nature. Its electromagnetic properties are extending from radio frequency and microwaves all the way up to optical frequencies^1^. It shows the electromagnetic properties due to both its cellular architecture and the chemical composition. Metamaterials unusual behaviors can be realized by applying certain artificial material structures rather than by using precise chemical compositions.

Electromagnetic (EM) properties of metamaterials are achieved mainly by proper designing of its unit cell. These properties (such as polarization conversion, magnetic permeability, magnetism, hyperbolic dispersion, refractive index close to zero, negative refractive index) cannot be found in nature^2^. Metamaterials can be categorized into resonant type and non-resonant type. The resonant type metamaterials can be further divided into three categories depending on permittivity and permeability functions; such as, zero-index materials, single negative metamaterials, and negative index metamaterial or double negative metamaterials^3^. Metamaterials are usually defined as artificial composite materials consisting of periodic unit cells. The tuneable negative permittivity and permeability of metacomposites can be achieved by tailoring their compositions and microstructures, which represents a new approach to material design^4,5^ applicable to natural composites^5,6^. The unique properties of metamaterials originate from their structures rather than compositions, and controlling both these parameters can enhance their electromagnetic characteristics^7,8^. Metal-based composites have attracted more attention than other composites. Some of these materials containing Ni, Fe, Ag, or Co particles randomly dispersed in a porous alumina host were prepared to obtain negative electromagnetic parameters in the radio frequency range^9,10^.

Because of its electromagnetic properties it has been widely used in several applications like energy harvesting^11^, space application^12^, filter design^13^, antenna design^14^, electromagnetic absorber^15^ etc. Recently, flexible materials have been considered for the preparation of metamaterials for the modern wireless systems due to their superior characteristics including light weight, ease of fabrication, low manufacturing costs, and high availability. They include liquid crystal polymers, liquid metals, polydimethylsiloxane, and various organic materials^16–18^. Moreover, Various types of metal aluminates (such as NiAl_2_O_4_, CoAl_2_O_4_) and ferrites (such as MnxZn(1-x)Fe_2_O_4_) are used to prepare such flexible materials^19,20^. Applications of such flexible materials are included in magnetic materials, pigments, sensors, catalysts, carriers, ceramic pigment, gas sensor, color TV tubes and solar absorber^21,22^. On the basis of components, the market is segmented into flexible displays, flexible battery, flexible sensors, flexible memory, photovoltaic and others. Flexible metamaterial technology, has its significance, generally in biomedical, cloaking and communication area, wherever the established rigid metamaterials cannot be brought up to the mark. The flexible material can be covered around any random shape to give the expected results.

Among the metal aluminates, Nickel aluminate (NiAl_2_O_4_) is a combined cation oxide with standard spinel assembly with general formula AB_2_O_4_, where “A” and “B” are divalent and trivalent cations, and related to Fd3m space group. In normal spinel, “A” occupy eight tetrahedral interstices, and “B” make up at sixteen octahedral sites. The NiAl_2_O_4_ is basically a binary oxide system (NiO-Al_2_O_3_) that have more than a few applications, for example: semiconductors, absorbents, and catalysts^23^. In pure form, NiO is p-type semiconductor and Al_2_O_3_ is a dielectric insulator. The NiAl_2_O_4_ is the mixed form of the NiO and Al2O3 and it shows some remarkable electrical performance differs from that of individual elements^24^. The synthesis procedure strongly influences the physical and dielectric properties of NiAl_2_O_4_. Several researchers synthesized crystalline NiAl_2_O_4_ using different methods like hydrothermal^25^, solid-state reaction^26^, mechano-chemical synthesis^27^, and sol-gel^28^, however it has not been explored much for microwave applications.

Islam *et al*.^29^ designed a metamaterial fabricated on a solid substrate with dimensions of 30 × 30 × 1.6 mm^3^ and negative refractive index bandwidth of 0.7 GHz. Hasan *et al*.^30^ reported a 10 × 10 mm^2^ ‘Z-shaped’ non-flexible resonator that was operational in the C- and X-bands and exhibited double negative characteristics. Liu *et al*.^31^ fabricated a single negative metamaterial on a solid FR4 substrate for X-band applications using a modified circular electric resonator. This material was relatively compact; however, its negative refractive index bandwidth was only 1 GHz. Ziolkowski *et al*.^32^ reported a non-planar solid substrate-based metamaterial with a bandwidth of only 900 MHz that exhibited double negative characteristics, whereas the negative refractive index bandwidth of the metamaterial synthesised on a flexible substrate was 4.02 GHz. Joshi *et al*.^33^ designed a flexible substrate-based metamaterial embedded into a wearable rectangular antenna that was operational only in the C-band of the microwave region and exhibited negative permeability in the frequency range from 8.35 GHz to 8.7 GHz. In general, the metamaterials with superior properties have been designed on solid substrates fabricated from silicon, Teflon, FR-4, Rogers, and Taconic materials.

In this work, NiAl_2_O_4_ crystals were synthesised using a sol-gel method to prepare flexible composite supports for metamaterials operational in the microwave frequency range. This method was utilised because of its various advantages including high homogeneity of the final product, good stoichiometric control, high productivity at low temperatures, and ability to produce unalloyed ultrafine powders. The dielectric properties of the synthesised NiAl_2_O_4_ substrates were carefully examined and discussed. Scanning electron microscopy (SEM), X-ray diffraction (XRD), and dielectric property analyses were performed to investigate the semiconducting behaviour, crystal structures, and microstructures of the produced materials. Impedance bandwidths as well as reflection coefficients, transmission coefficients, dielectric permittivities, magnetic permeabilities, and refractive indices were measured to confirm the suitability of the prepared flexible substrates for microwave applications. Furthermore, metamaterial structures were also fabricated using the produced NiAl_2_O_4_ materials as flexible substrate, and their reflection (S_11_) and transmission (S_21_) coefficients were determined using the commercially available CST Microwave Studio electromagnetic simulator software.

## Materials and Methods

### Preparation of NiAl_2_O_4_-based flexible substrates

**Figure 1:**
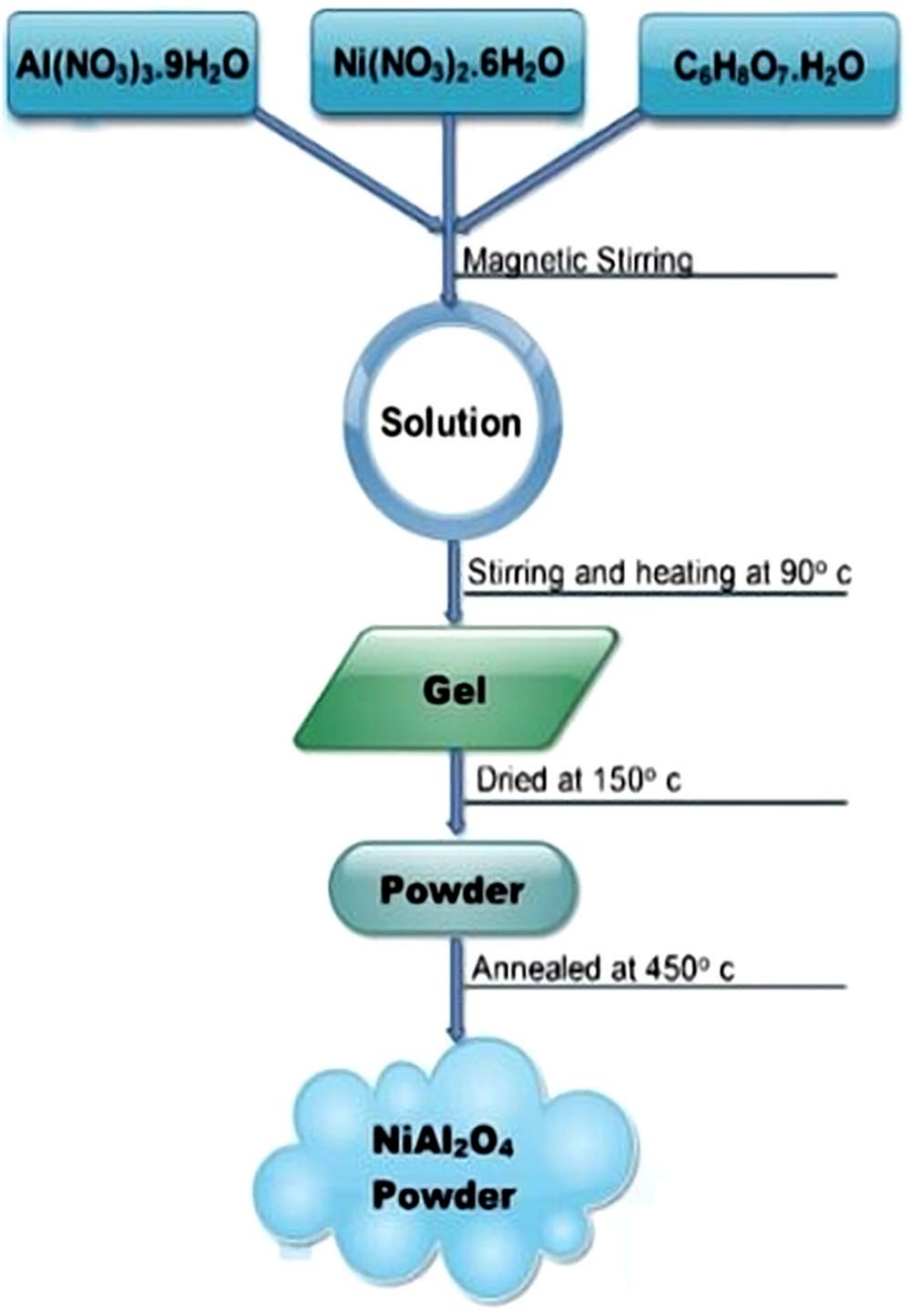
Flowchart for Nickel aluminate (NiAl_2_O_4_) compound preparation.

The entire synthesis process of NiAl_2_O_4_ nano-powder is described in Fig. 1. Al(NO_3_)_3_ 3.9H_2_O and Ni(NO_3_)_2_ 2.6H_2_O are utilised as raw materials mixed at molar ratios of 0.36 (Ni): 0.64 (aluminium nitrate) (labelled as Ni_36_) and 0.42 (Ni): 0.58 (aluminium nitrate) (labelled as Ni_42_) and subsequently dissolved in distilled water using citric acid as a chelating agent. As a result, a semi-transparent light green viscous solution is formed. NiAl_2_O_4_ samples are prepared at a lower temperature via an appropriate citrate sol-gel method with an admirable mechanism over the stoichiometry and easier dopant outline. To evaporate water from the resulting solution, the latter is heated to 90 °C and stirred continuously for about 4 h to produce a greenish gel, which is subsequently transferred into an alumina crucible and placed inside a furnace heated to 150 °C for 2 h to complete the chemical reaction. The obtained precursor is ground into fine powder and calcined at 450 °C for 1 h. At the same time, polyvinyl acetate (PVA) glue binder is prepared by dissolving a specified amount of PVA in distilled water.

**Figure 2:**
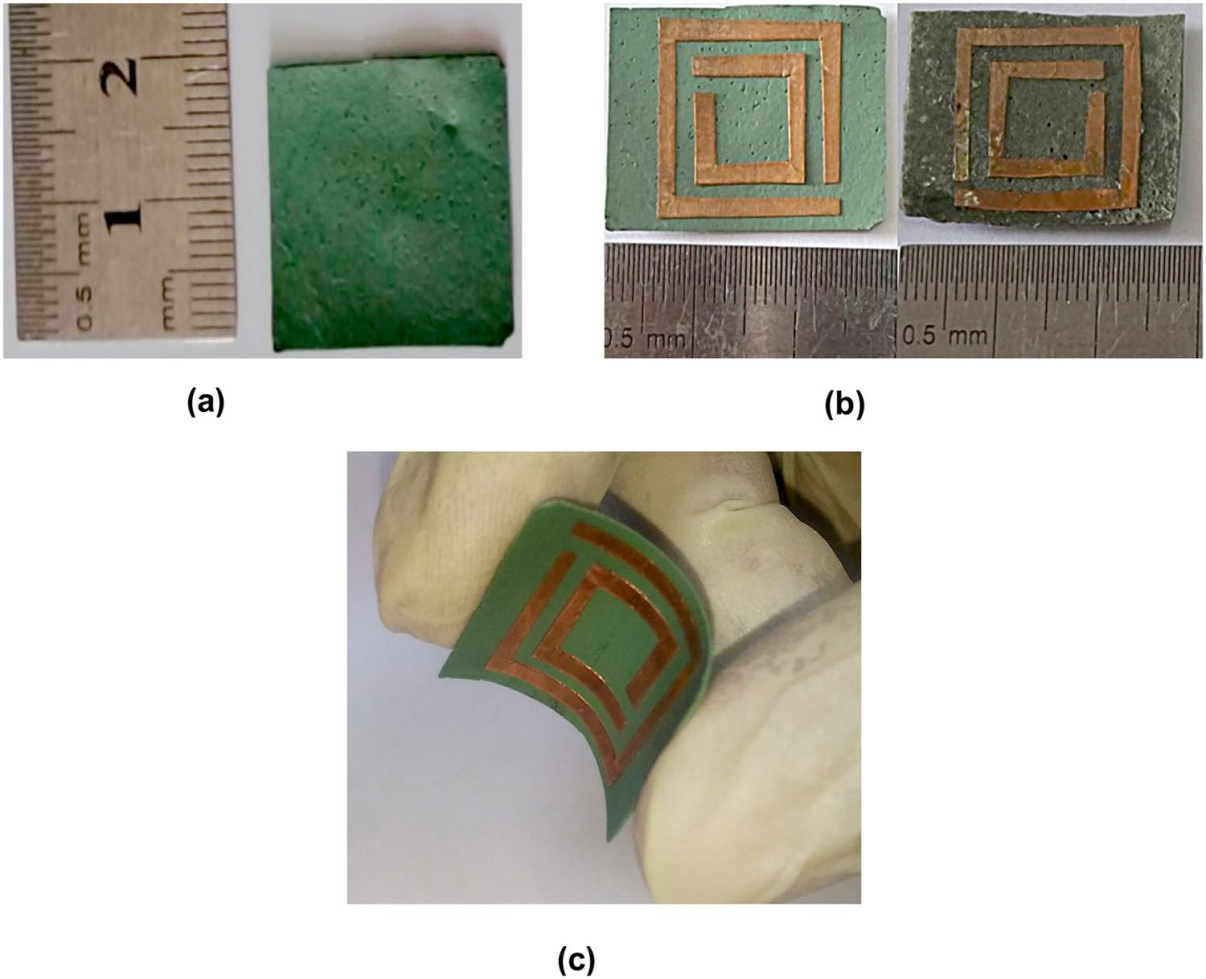
(**a**) A fabricated Ni_36_ flexible substrate. (**b**) N_36_ and N_42_ flexible substrates containing resonators for metamaterial applications. (**c**) Flexibility characterisation.

Figure 2(a,c) show the standard and twisting situations of the flexible substrates, fabricated by adding the synthesized NiAl_2_O_4_ powder to the PVA solution at a ratio of 1 g to 10 mL followed by stirring. In Fig. 2(b) two fabricated substrate is shown. The crystal structure and phase composition are examined by using XRD (model: Siemens D 500; Cu Kα).

The surface morphology is examined by SEM (Carl Zeiss Supra). The loss tangent and dielectric constant is measured over a frequency range of 1–8 GHz at room temperature. Their relative permittivities and dielectric loss tangents are measured in the frequency range from 200 MHz to 20 GHz using a DAK dielectric measurement kit. The obtained permittivity values are equal to 4.94 (Ni_36_) and 4.97 (Ni_42_), whereas the corresponding loss tangents are 0.01 (Ni_36_) and 0.007 (Ni_42_).

### Metamaterial preparation and related methodology

**Figure 3:**
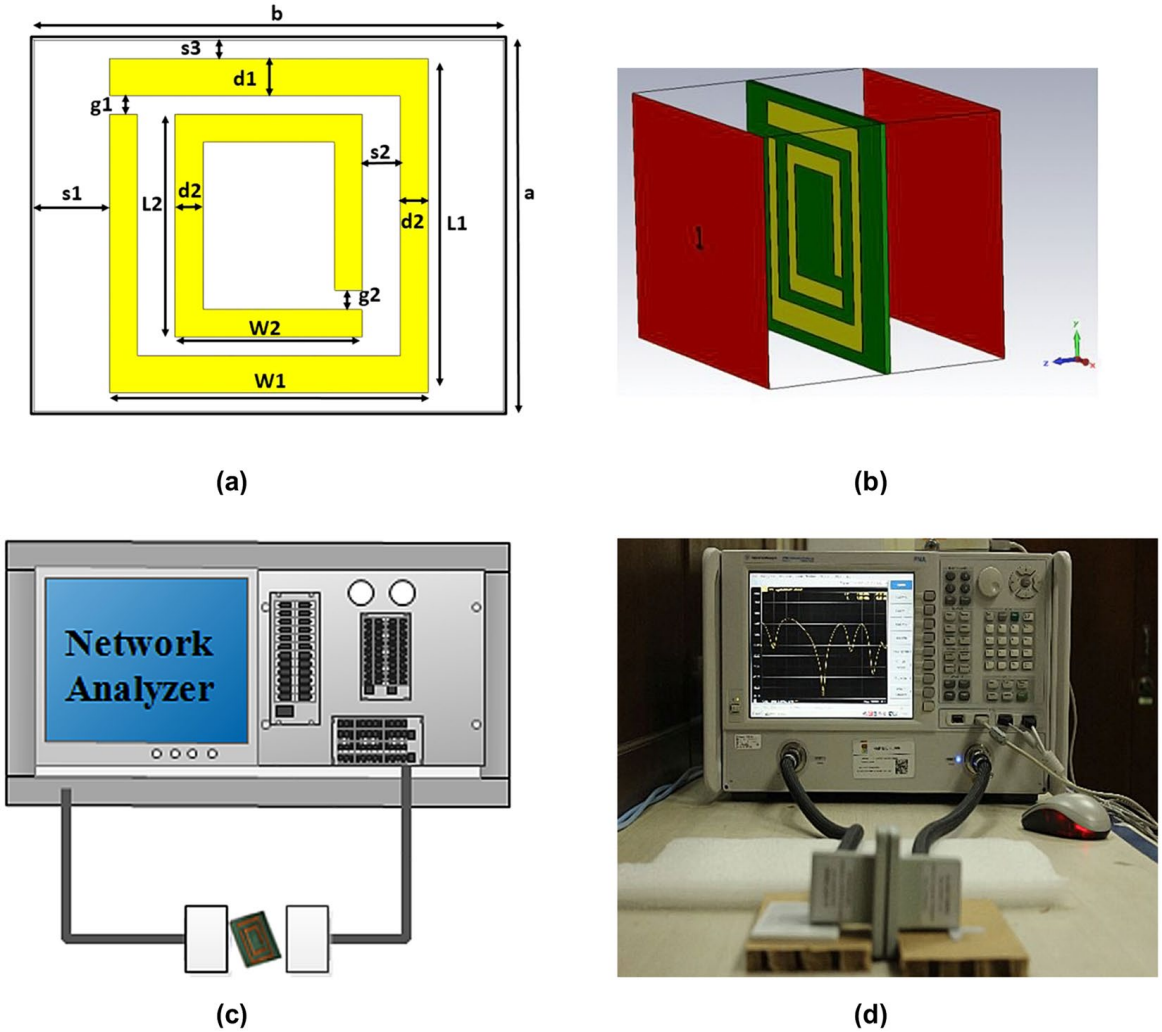
(**a**) Metamaterial unit cell layout, (**b**) Simulated geometry for wave propagation (**c,d**) Measurement setup for metamaterial characteristics test.

To verify the proposed substrate for its metamaterial characteristics the split ring resonators are used. A schematic view of the unit cell is shown in Fig. 3(a). The unit cell 25 × 20 mm^2^ has outer and inner ring resonator which is made of copper with a thickness of 0.035 mm. In this design two different types of nickel aluminate (NiAl_2_O_4_) based flexible substrates are used with molar ratio 0.36:0.64 (Ni_36_) and 0.42:0.58 (Ni_42_) for nickel and aluminum. Both of the cases the substrate thickness were 0.50 mm. All the design parameters and dimensions are represented in Table 1 where, a is the substrate length, b is the substrate width, L1 is the outer ring resonator length, W1 is the ring resonator outer ring resonator width, L2 inner ring resonator length,W2 is the inner ring resonator width, s1 is the distance between the outer ring and the length edge of the substrate, s2 is the gap among the outer ring and inner ring resonator, s3 is the distance between the outer ring and the width edge of the substrate, d1 is the metal strip width of one side of the ring resonator, d2 is the metal strip width of the inner ring and outer ring other side of the ring resonator and g1, g2 are the gaps between the outer and inner ring resonator. Figure 3(b) represent the simulated geometry of wave propagation in the CST microwave studio. Measurement set up for finding metamaterial characteristics is shown in Fig. 3(c,d).

**Table 1:** Unit cell specifications.

Design parameter	Dimension(mm)	Design parameter	Dimension(mm)
a	20	s2	2
b	25	s3	1
L1	18	d1	2
L2	12	d2	1.50
W1	17	g1	1
W2	10	g2	1
s1	4		

A commercially available finite integration technique based electromagnetic simulator CST Microwave Studio is used to find scattering parameter and to calculate the effective medium parameters. The incident electromagnetic wave applied in the z direction by the two waveguide ports. Perfect electric conductor (PEC) and perfect magnetic conductor (PMC) are applied in x and y direction respectively. The tetrahedral mesh with 50Ω normalized impedance and frequency domain solver is used for the simulation of 2 to 12 GHz. For utilizing the metamaterial characteristics two split ring resonator is used where split gaps are created capacitance and metal strip are for inductance forming.

The fabricated metamaterial prototypes are positioned between two microwave waveguide ports (four different waveguide ports are utilised in this work). Measurements are conducted using a vector network analyser (VNA; Agilent N5227A, CA, USA) calibrated by an Agilent N4694-60001 electric calibration module (the entire measurement setup is depicted in Fig. 3(c,d)). The Nicolson-Ross-Weir method (NRW) is used to calculate the effective permittivities, magnetic permeabilities, and refractive indices of the metamaterials^28^.

## Results and Discussion

### Flexible material characterisation

Average crystalline sizes of the synthesised materials are determined, and the formation of the spinel structure is confirmed using SEM, XRD, and EDX techniques as well as by measuring their dielectric constants, loss tangents, and conductivities.

### XRD measurements

**Figure 4:**
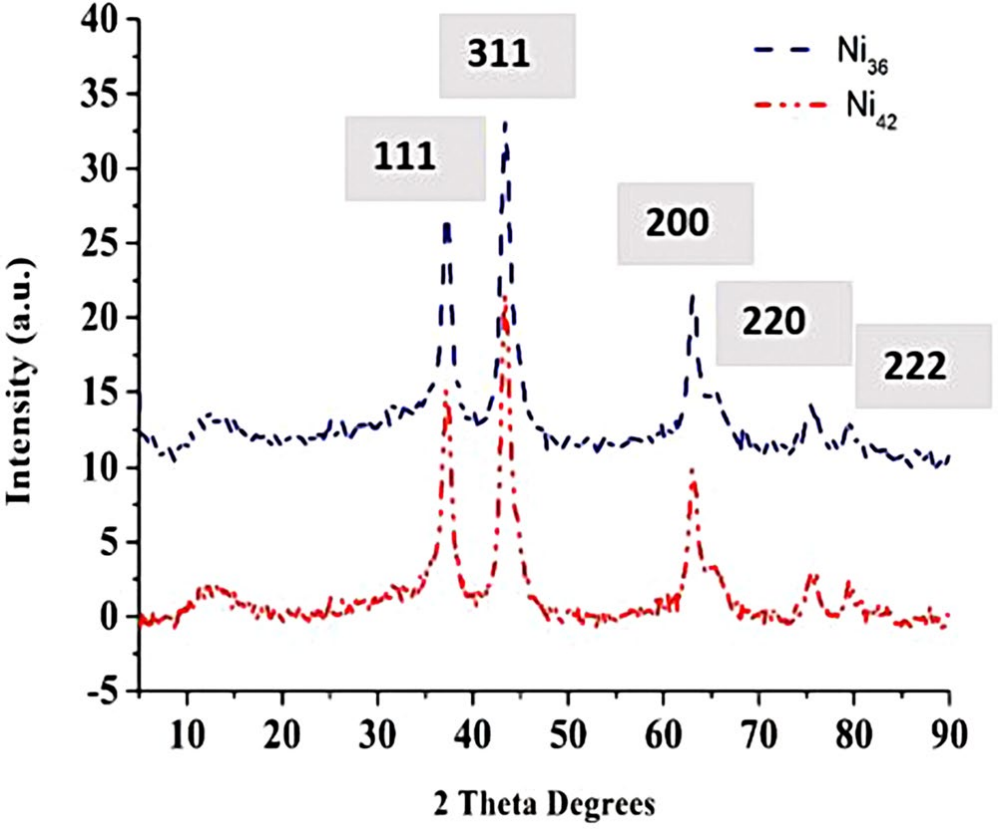
XRD patterns of the prepared nickel aluminate (NiAl_2_O_4_) sample.

The main phases of the prepared Ni-based catalysts have been identified using a Siemens D500 X-ray diffractometer. The utilised X-ray radiation source contains a Cu Kα anode (40 kV, 20 mA), and the magnitudes of 2θ range between 5° and 90°. The XRD spectra recorded for the manufactured samples are shown in Fig. 4. Two major diffraction peaks are shown in Fig. 4. They are identified and indexed as the typical cubic spinel structure of NiAl_2_O_4_ (Pattern No. PDF-01-078-1601) and NiO (Pattern No. PDF-00-001-1239) with a leading peak (311) and (200) for both Ni_36_ and Ni_42_ respectively. Diffraction peaks have been seen at 2θ = 37° (111), 45° (311), 63° (200), 75° (220), 80° (222).

The average crystallite size of NiAl_2_O_4_ is calculated from the line width of the (311) reflection using Scherrer’s equation:

1 $${\rm{D}}=\frac{0.94\,{\rm{\lambda }}}{{\rm{\beta }}\,\cos \,{\rm{\theta }}},$$

where λ is the X-ray wavelength (1.54060 nm), β is the broadening of the diffraction line corresponding to the half value at the maximum intensity (in radians), and θ is the diffraction angle. The calculated crystallite size of NiAl_2_O_4_ is 13 nm, which is very close to the tabulated value of 12 nm^34^. The lattice parameter of NiAl_2_O_4_ is determined from the positions of the XRD peaks using the following equation:

2 $${\rm{a}}={\rm{d}}\sqrt{{h}^{2}+{k}^{2}+{l}^{2}},$$

where d = 2.42754 nm is the inter-planar distance, and the (hkl) Miller indices of the leading peak are represented by the notation (311). The calculated lattice parameter of NiAl_2_O_4_ is consistent with the tabulated value of a = 8.048 Å^34^.

### SEM and EDX studies

**Figure 5:**
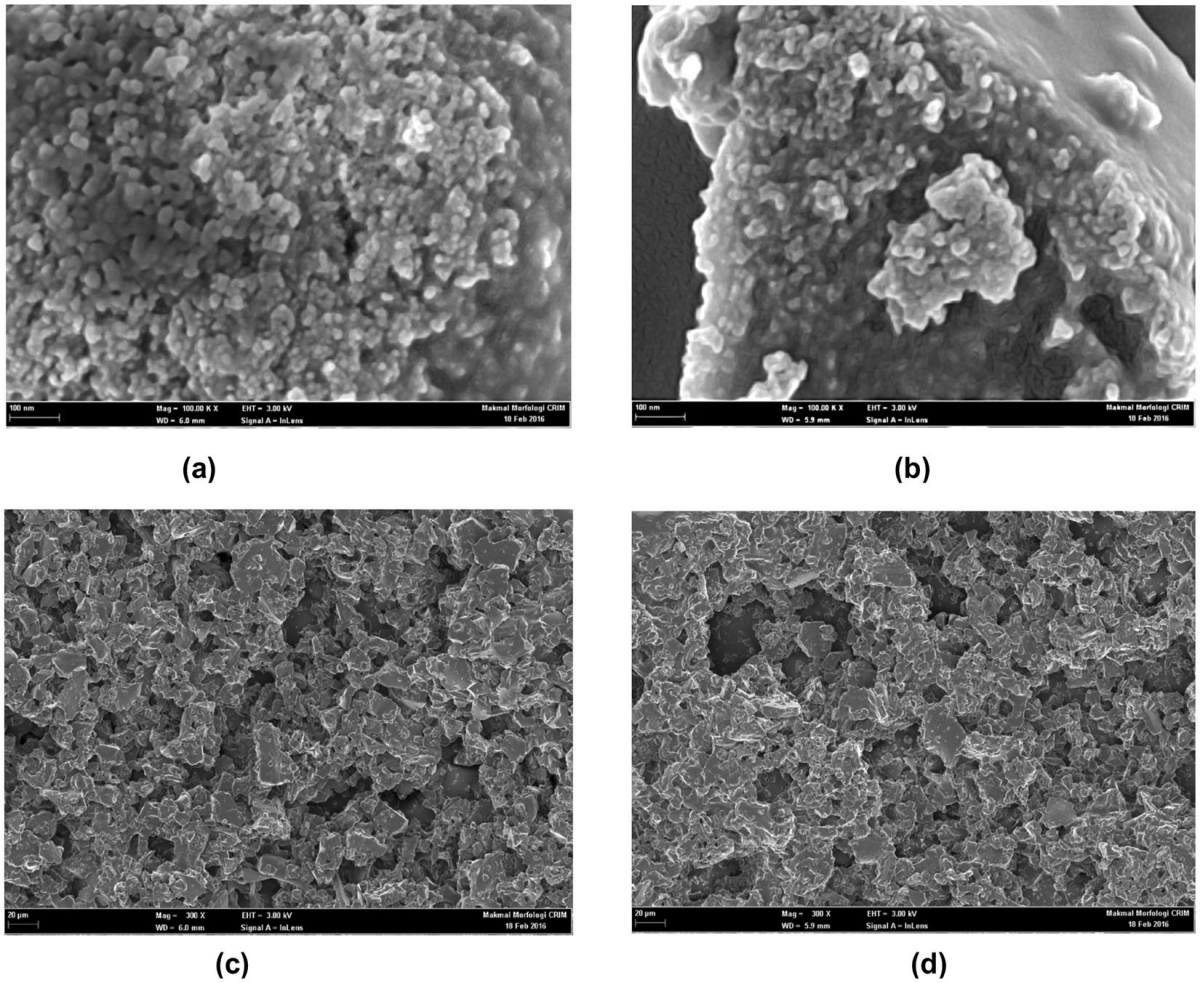
SEM images of the NiAl_2_O_4_ samples obtained at magnifications the lengths of the corresponding scale bars (**a**) 100 nm (Ni_36_), (**b**) 100 nm (Ni_42_), (**c**) 20 µm (Ni_36_), and (**d**) 20 µm (Ni_42_).

The SEM and EDX techniques are used to analyse the composite morphology and composition. The SEM images of the Ni_36_ and Ni_42_ thin films obtained at different magnifications are shown in Fig. 5. In general, annealing the prepared specimens increases their average grain sizes. Furthermore, the average grain size of NiAl_2_O_4_ particles is equal to 13 nm, which is close to the value calculated from the results of XRD analysis; however, increasing the Ni content increases the grain size up to 16 nm and decreases the material porosity, indicating that the bigger grains correspond to pure Ni, while the smaller ones - to NiAl_2_O_4_ (normally, increasing the composite grain size significantly improves its quality and dielectric constant).

**Figure 6:**
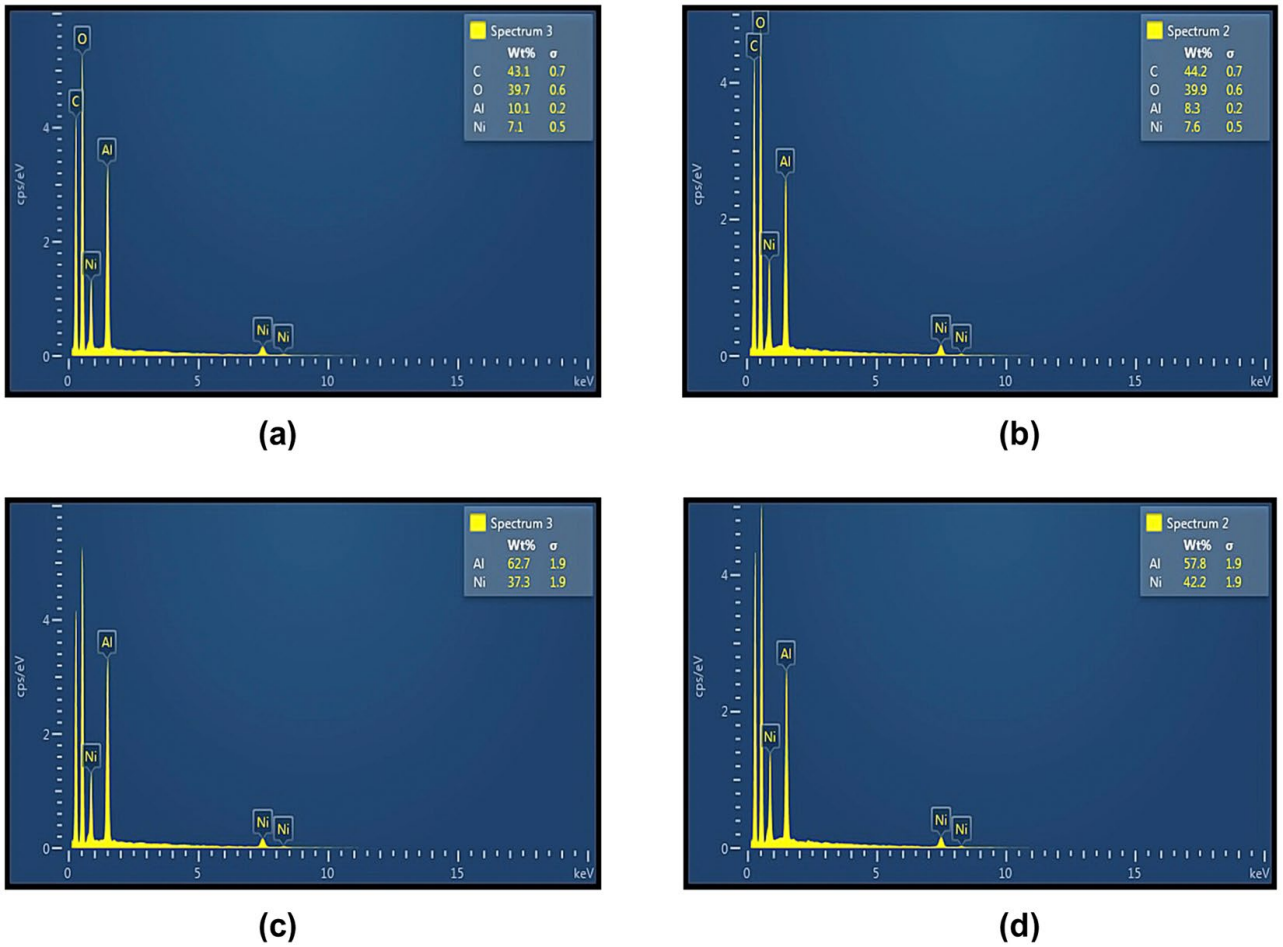
EDX patterns obtained for the Ni_36_ and Ni_42_ samples by considering (**a**) and (**c**) all elements and (**b**) and (**d**) only nickel and aluminium elements.

The produced NiAl_2_O_4_ nano-powders contain spherical particles with homogeneous distribution as shown in Fig. 5(a,b) for Ni_36_ and Ni_42_ (the corresponding images obtained at larger magnifications are displayed in Fig. 5(c,d), respectively). The compositions of the prepared specimens are determined via EDX measurements, and the contents of C, O, Ni, and Al elements in the Ni_36_ and Ni_42_ specimens are shown in Fig. 6(a,b), respectively (Fig. 6(c,d) display the relative amounts of nickel and aluminium elements in the surface layers of the fabricated samples). The relative mass contents of Ni and Al in the Ni_36_ specimen are equal to 37.3% and 62.7%, which are rounded to 36% and 64%, respectively (Fig. 6(c)), while for the Ni_42_ sample, their magnitudes are equal to 42.2% and 57.8% (42% and 58% in Fig. 6(d), respectively) at the early stage of the sol-gel synthesis process. The corresponding atomic ratios of nickel and aluminium are equal to 1:2 for Ni_36_ and 2:3 for Ni_42_. On the other hand, the atomic ratio between the metal and non-metal elements is also 1:2, indicating that the synthesised NiAl_2_O_4_ compounds can be used as substrates for metamaterials.

### Dielectric permittivity

**Figure 7:**
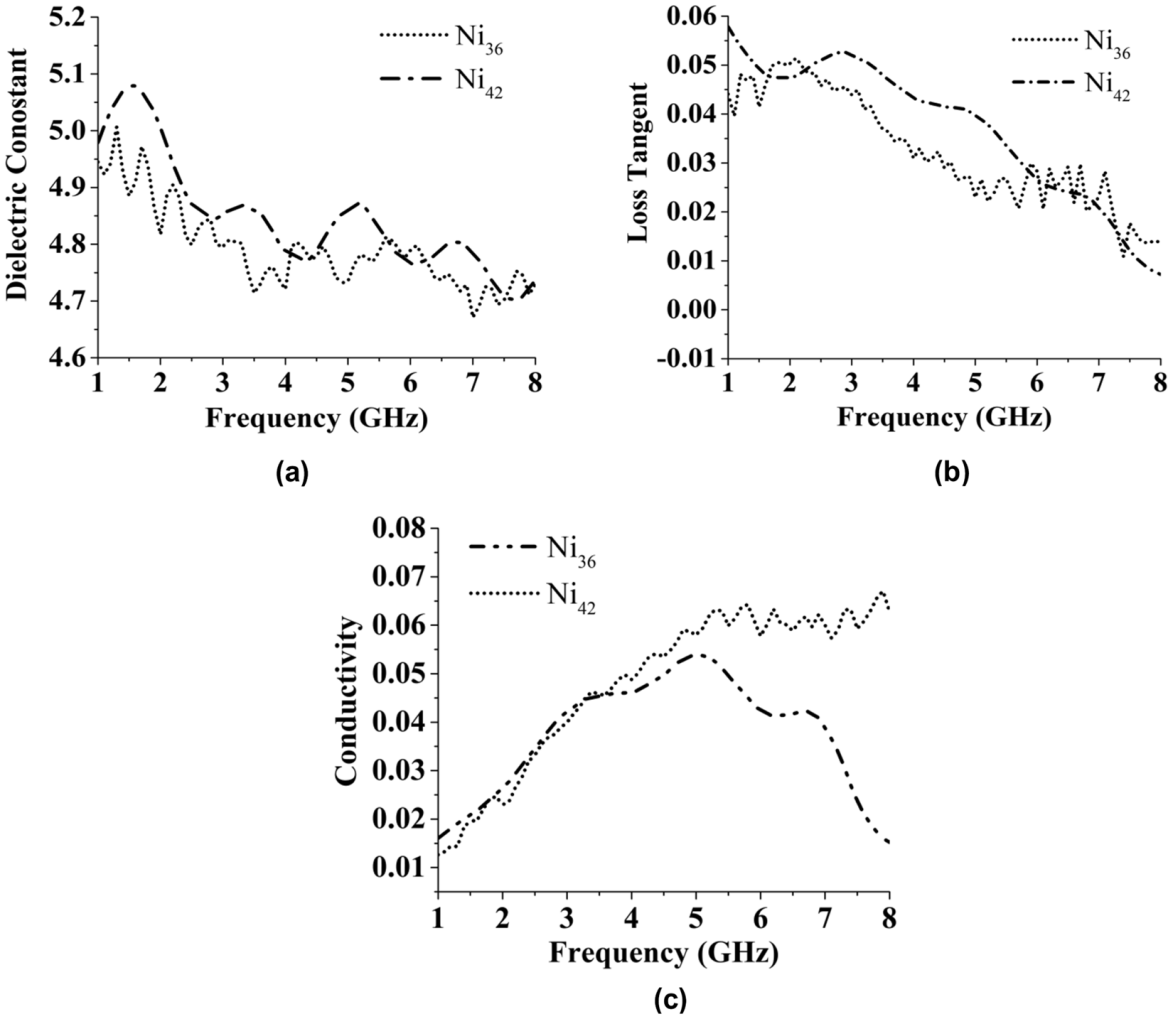
(**a**) Dielectric constant against frequency and (**b**) loss tangent and (**c**) Conductivity against the same frequency range of the prepared NiAl_2_O_4_ substrate.

The utilised DAK dielectric measurement kit is typically utilised to determine the dielectric constants and loss tangents of various materials in the frequency range between 200 MHz and 20 GHz range using a single open-ended coaxial dielectric probe. Its software contains advanced algorithms for high-precision and fast measurements of dielectric parameters, including the real and imaginary parts of conductivity (σ), dielectric constant (ε′& ε″), and loss tangent (tan δ) and processing data structures in numerous forms such as linear and logarithmic charts, Cole-Cole plots, and Smith charts. Figure 7(a) shows the values of dielectric constants obtained for the Ni_36_ and Ni_42_ samples, and Fig. 7(b) depicts the loss tangents of the synthesised substrate materials plotted in the frequency range of 1−8 GHz. The relative permittivity of the substrate fabricated from NiAl_2_O_4_ powder decreases with an increase in the operational frequency, which is consistent with Koop’s phenomenological hypothesis and the Maxwell-Wagner model of interfacial polarization^19,20^. According to the Debye model, the reduction in the loss tangent with increasing frequency results from interfacial polarization, which offers go up to an unwinding procedure with a long unwinding time (as compared with the electronic or dipolar polarization). The dielectric constant *ε*_𝑟_ of Ni_36_ is 4.94, while that of Ni_42_ is equal to 4.97. Normally, the variations of *ε*_𝑟_ values obey the following logarithmic mixing rule:

3 $$\mathrm{ln}\,{\varepsilon }_{r}=v1\,\mathrm{ln}\,{\varepsilon }_{r}1+v2\,\mathrm{ln}\,{\varepsilon }_{r}\,2$$

where *ε*_𝑟_1 and *ε*_𝑟_2 are the dielectric constants of the phases with volumes ν1 and ν2, respectively. The densities and dielectric constants increase with increasing Ni content, while the value of the loss tangent decreases from 0.01 (Ni_36_) to 0.007 (Ni_42_). The observed increases in the composite density and dielectric constant originate from the increase in the grain size (Fig. 5). Figure 7(a) also shows that the variations of *ε*_𝑟_ are consistent with the density variations, suggesting that the *ε*_𝑟_ values of NiAl_2_O_4_ materials are mainly controlled by their apparent density. Therefore, it can be concluded that the synthesised NiAl_2_O_4_ composites exhibit low dielectric constants (*ε*_𝑟_ < 15) and thus are good candidate materials for microwave applications.

Figure 7(c) displays the variations in conductivity measured at different sample compositions. It shows that higher conductivities are observed at higher frequencies confirming the existence of polaron hopping in the studied samples. In general, electrical conductivity increases with frequency; however, at low concentrations and certain temperatures, its value begins to decrease after 5 GHz. The real ac conductivity *σ*_ac_ consists of the following two parts:

4 $${\sigma }_{ac}=\sigma (T)+\sigma (\omega )$$

The first term *σ* (*T*), which is frequency and temperature dependent, results from the drift mobility of electric charge carriers, whereas the second term *σ* (*ω*) is related to the dielectric relaxation caused by the localized electric charge carriers.

### Flexible substrates: metamaterial behaviour

The two prepared flexible substrate Ni_36_ and Ni_42_ exhibit metamaterial behaviour that is analysed below.

### Metamaterial characteristics of Ni_36_

**Figure 8:**
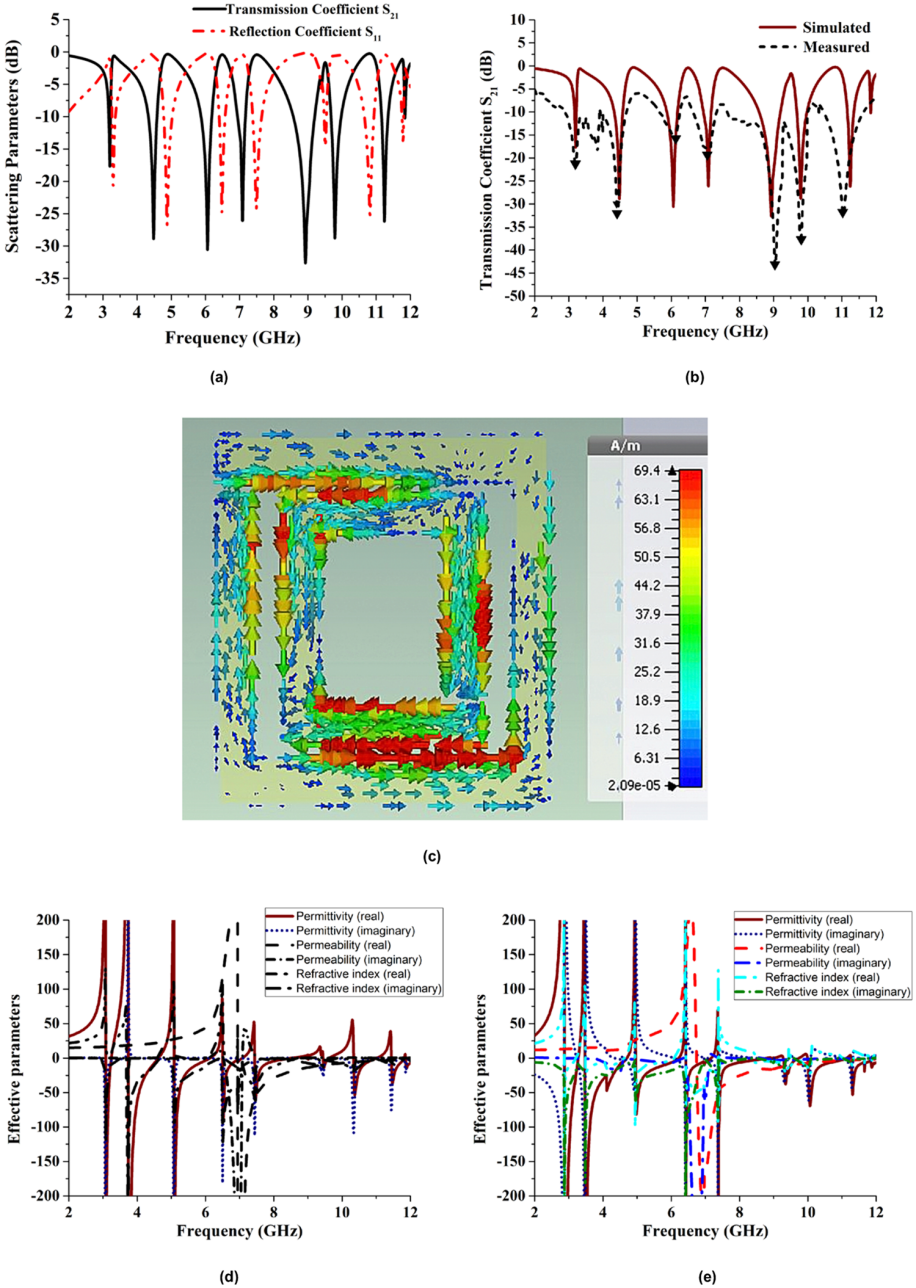
Characteristics of the Ni_36_ flexible substrate: (**a**) simulated transmission (S_21_) and reflection (S_11_) coefficients, (**b**) measured and simulated values of the transmission coefficient S_21_, (**c**) surface current distribution at a frequency of 7.42 GHz, (**d**) simulated effective parameters, and (**e**) measured effective parameters.

The simulated reflection (S_11_) and transmission (S_21_) coefficients of the fabricated flexible substrate Ni_36_ are depicted in Fig. 8(a), whereas Fig. 8(b) contains the simulated and measured values of the transmission coefficient S_21_. The obtained magnitudes are as follows: 3.19 GHz (S-band), 4.48 GHz (C-band), 6.05 GHz (C-band), 7.08 GHz (C-band), 8.92 GHz (X-band), 9.79 GHz (X-band), and 11.24 GHz (X-band). Furthermore, the measured maximum resonance frequencies are equal to 3.18 GHz (S-band), 4.42 GHz (C-band), 6.11 GHz (C-band), 7.10 GHz (C-band), 9.05 GHz (X-band), 9.78 GHz (X-band), and 11.03 GHz (X-band). Due to fabrication or measurement errors, the measured numerical and experimental frequencies slightly differ from each other; however, all the obtained values are below −10 dB.

The surface current distribution in the unit cell determined at a frequency of 7.42 GHz is shown in Fig. 8(c). Two dominating current paths are observed in the two split gap areas. The arrows denote the directions of the currents in the outer and inner ring resonators, and the colours represent their intensities. Since the surface currents in the outer and inner ring resonators flow in the opposite directions, they nullify each other in a resulting stop band (the intensities of the currents clearly indicate the overall current direction). Actually, the two currents are antisymmetric at the resonance and form a loop, which can be characterised by an equivalent magnetic dipole moment, which creates artificial magnetic properties of the studied structure, leading to its negative effective permeability.

Figure 8(d) shows the simulated effective parameters of the fabricated N_36_ substrate, and Fig. 8(e) describes its VNA-measured effective parameters analysed by the NRW method. In particular, Fig. 8(d) displays the negative values of the effective permittivity determined from the simulated scattering parameters in the frequency ranges from 3.08 to 3.24 GHz (bandwidth: 0.16 GHz), 3.74 to 4.62 GHz (bandwidth: 0.88 GHz), 5.08 to 6.13 GHz (bandwidth: 1.05 GHz), 6.51 to 7.07 GHz (bandwidth: 0.56 GHz), 7.45 to 8.53 GHz (bandwidth: 1.08 GHz), 9.45 to 9.62 GHz (bandwidth: 0.17 GHz), 10.34 to 11.01 GHz (bandwidth: 0.67 GHz), 11.45 to 11.81 GHz (bandwidth: 0.36 GHz), and 11.86 to 12 GHz (bandwidth 0.14 GHz).

Moreover, according to Fig. 8(d), the magnetic permeability exhibits a bandwidth of 5.05 GHz (from 6.95 to 12 GHz). At higher frequencies, the generated current is unable to adjust to the electromagnetic force, producing a time lag. Due to polarization, the dielectric permittivity and magnetic permeability of a fabricated substrate depend on its internal structure. The obtained values of the refractive index of Ni_36_ are shown in Fig. 8(d). The corresponding frequency ranges span from to 3.69 to 4.25 GHz (bandwidth: 0.56 GHz), 5.23 to 5.91 GHz (bandwidth: 0.68 GHz), and 6.59 to 12 GHz (bandwidth: 5.41 GHz). When the permittivity, permeability, and refractive index of a metamaterial are negative, the latter is called a double negative metamaterial. For the N_36_ substrate, the values of the dielectric permittivity (−60.19), magnetic permeability (−56.04), and refractive index (−65.60) measured at a frequency of 7.46 GHz are negative (as well as the magnitudes obtained at a frequency of 11.88 GHz and equal to −15.24, −2.43, and −6.61, respectively). Therefore, the corresponding unit cell can be called a left-handed metamaterial at frequencies of 7.46 and 11.88 GHz.

Figure 8(e) displays the negative values of the effective dielectric permittivity determined from the measured scattering parameters. Their frequency ranges span from 2.87 to 3.27 GHz (bandwidth: 0.40 GHz), 3.48 to 4.7 GHz (bandwidth: 1.22 GHz), 4.96 to 6.21 GHz (bandwidth: 1.25 GHz), 6.43 to 7.17 GHz (bandwidth: 0.74 GHz), 7.83 to 8.82 GHz (bandwidth: 0.99 GHz), 9.29 to 9.74 GHz (bandwidth: 0.45 GHz), 9.99 to 11.13 GHz (bandwidth: 1.14 GHz), and 11.27 to 12 GHz (bandwidth: 0.73 GHz). It also shows that the obtained permeability values exhibit a bandwidth from 6.76 to 12 GHz. The same figure depicts the negative refractive indices in the ranges from 2.87 to 3.24 GHz (bandwidth: 0.37 GHz), 3.49 to 4.62 GHz (bandwidth: 1.13 GHz), 4.94 to 6.09 GHz (bandwidth: 1.15 GHz), 6.43 to 7.41 GHz (bandwidth: 0.98 GHz), 8.45 to 10.01 GHz (bandwidth: 1.56 GHz), and 10.97 to 11.33 GHz (bandwidth: 0.36 GHz). In addition, the dielectric permittivity (−66.09), magnetic permeability (−54.77), and refractive index (−62.45) of the Ni_36_ substrate measured at a frequency of 7.43 GHz are negative (their corresponding values determined at 11.35 GHz are equal to −70.54, −43.67, and −56.98, respectively). Hence, the Ni_36_ unit cell can be called a left-handed metamaterial at frequencies of 7.41 GHz and 11.33 GHz.

### Metamaterial characteristics of Ni_42_

**Figure 9:**
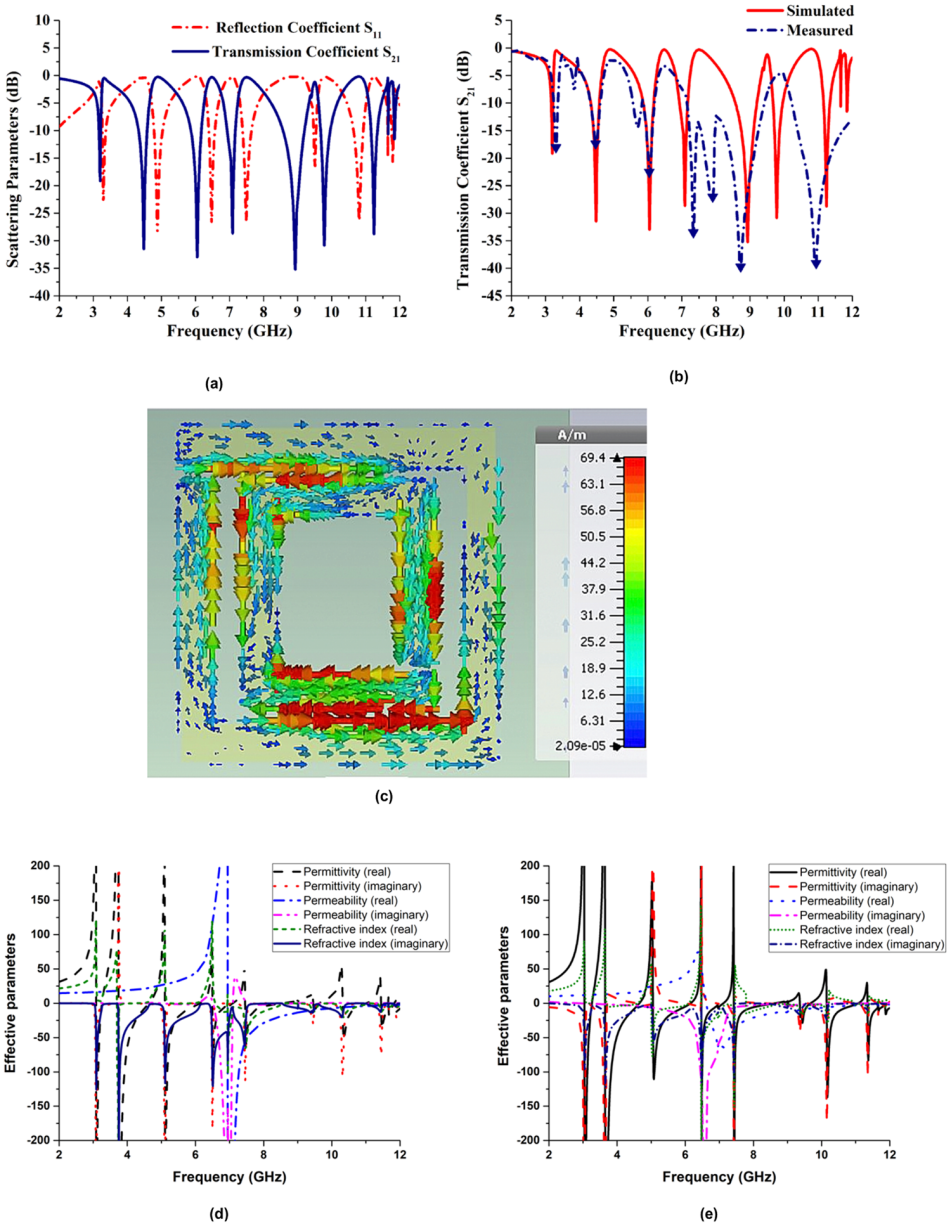
Characteristics of the Ni_42_ flexible substrate: (**a**) simulated transmission (S_21_) and reflection (S_11_) coefficients, (**b**) measured and simulated values of the transmission coefficient S_21_, (**c**) surface current distribution at a frequency of 7.42 GHz, (**d**) simulated effective parameters, and (**e**) measured effective parameters.

The scattering parameters obtained for the Ni_42_ substrate are shown in Fig. 9(a), while Fig. 9(b) contains the simulated and measured values of the transmission coefficient S_21_. The simulated maximum resonance frequencies of Ni_42_ are equal to 3.20 GHz (S-band), 4.48 GHz (C-band), 6.05 Hz (C-band), 7.09 GHz (C-band), 8.93 GHz (X-band), 9.78 GHz (X-band), and 11.24 GHz (X-band), whereas their measured magnitudes are 3.30 GHz, 4.50 GHz, 6.05 GHz, 7.36 GHz, 8.72 GHz, and 10.91 GHz, respectively.

The surface current distribution for Ni_42_ at 7.43 GHz is shown in Fig. 9(c). Here also two dominating current path are created in the two split gap areas and the surface currents in the outer and inner ring resonators also flow in the opposite directions. Figure 9(d) shows the simulated effective parameters of the fabricated N_42_ substrate, and Fig. 9(e) describes its VNA-measured effective parameters analysed by the NRW method. In particular, Fig. 9(d) shows the negative values of the effective permittivity determined from the simulated scattering parameters in the frequency ranges from 3.08 to 3.24 GHz (bandwidth: 0.16 GHz), 3.74 to 4.62 GHz (bandwidth: 0.88 GHz), 5.09 to 6.12 GHz (bandwidth: 1.03 GHz), 6.50 to 7.08 GHz (bandwidth: 0.58 GHz), 7.45 to 8.53 GHz (bandwidth: 1.08 GHz), 9.44 to 9.62 GHz (bandwidth: 0.18 GHz), 10.33 to 11.01 GHz (bandwidth: 0.68 GHz), 11.44 to 11.81 GHz (bandwidth: 0.37 GHz) and 11.87 to 12 GHz (bandwidth: 0.13 GHz). Moreover, according to the Fig. 9(d) permeability exhibits a bandwidth 6.95 GHz (from 6.95 to 12 GHz). The obtained values of the refractive index is shown in the Fig. 9(d). It shows negative refractive index frequency ranges are from 3.62 to 4.31 GHz (bandwidth: 0.69 GHz), 5.19 to 5.94 GHz (bandwidth: 0.75 GHz), 6.57 to 8.51 GHz (bandwidth: 1.94 GHz), 8.7 to 12 GHz (bandwidth: 3.30 GHz). For the Ni_42_ substrate, the dielectric permittivity, magnetic permeability and refractive index negative in 7.47 GHz and 11.89 GHz. Therefore, the corresponding unit cell can be called a left-handed metamaterial at frequencies of 7.47 and 11.89 GHz.

In Fig. 9(e) displays the negative values of the measured effective dielectric permittivity that is determined from the scattering parameters. The frequency ranges span from 3.04 to 3.26 GHz (bandwidth: 0.22 GHz), 3.65 to 4.65 GHz (bandwidth: 1 GHz), 5.05 to 6.15 GHz (bandwidth: 1.1 GHz), 6.48 to 7.11 GHz (bandwidth: 0.63 GHz), 7.43 to 8.63 GHz (bandwidth: 1.2 GHz), 9.37 to 9.67 GHz (bandwidth: 0.3 GHz), 10.15 to 11.07 GHz (bandwidth: 0.92 GHz), 11.35 to 12 GHz (bandwidth: 0.65 GHz). Moreover, the obtained permeability values exhibit a negative range bandwidth 6.62 to 12 GHz. The same figure represents the refractive indices in the range from 3.11 to 3.19 GHz (bandwidth: 0.08 GHz), 3.69 to 4.61 GHz (bandwidth: 0.92 GHz), 5.01 to 6.1 GHz (bandwidth: 1.09 GHz), 6.47 to 10.21 GHz (bandwidth: 3.74 GHz). In addition, the dielectric permittivity, magnetic permeability and refractive index are negative in 7.43 GHz and 11.35 GHz. Therefore, the proposed metamaterial can be called left-handed metamaterial on that two frequency.

Ni_36_ is more porous than Ni_42_; as a result, Ni_42_ exhibits a higher dielectric constant. As the nickel content in the fabricated nickel aluminate substrate increases, its dielectric constant also increases, while the magnitude of the tangent loss decreases. The value of the dielectric constant depends on the average grain size, cation distribution, sintering temperature, and content of nickel ions. Furthermore, the effect of the nickel content on the resonance frequency of the fabricated nickel aluminate composites is observed as well (its magnitude increases with increasing nickel content together with the average grain size, which reduces the material porosity). However, both metamaterials synthesised in this study demonstrate double negative characteristics. Table 1 lists the parameters of the Ni_36_ and Ni_42_ metamaterials, whereas their comparison with the literature values is performed in Table 2.

**Table 2:** Technical specification of the proposed substrate based metamateril for Ni36 and Ni42.

Substrate types	Dielectric constant	DNG Frequency (GHz)	Metamaterial type	Effective medium ratio($\frac{{\boldsymbol{\lambda }}}{{\boldsymbol{b}}}$)	Loss tangent	Maximum Conductivity (S/m)	Grain size (nm)
Ni_36_	4.94	7.46	DNG	4.15	0.01	0.05805	13
		11.88					
Ni_42_	4.97	7.47	DNG	4.16	0.007	0.06703	16
		11.89					

It should be noted that the designed metamaterial substrates are flexible ones with a bandwidth for the negative refractive index equal to 5.41 GHz (see Table 3). Both of them are applicable for the S-, C-, and X-bands, and their effective medium ratios are greater than 4. Therefore, these two substrates are compact, flexible, and cost-effective materials. Joshi *et al*. reported a flexible substrate that was applicable only for the C-band and possessed a bandwidth for the negative refractive index equal to 350 MHz, whereas the negative bandwidth for the metamaterial substrate proposed in this work is 5.41 GHz. In addition, the same authors fabricated a flexible substrate with single negative metamaterial characteristics, while the substrates described in this study possess double negative metamaterial characteristics. In other works, solid substrates were typically used, and their negative refractive indices were smaller than those of the Ni_36_ and Ni_42_ metamaterials. On the other hand, Qu *et al*.^35^ designed a SiO_2_-coated Fe particles fabricated by a modified Stober method for Fe/epoxy percolated composites. The electromagnetic properties of the produced composites were investigated in the radio frequency range, and their negative permittivity values were adjusted by the invariant permeability to obtain double negative characteristics. Metallic fillers and carbonaceous materials are usually utilised for designing metacomposites; however, various ceramic fillers (such as titanium nitride) are also used for this purpose. Because of its high temperature durability, good chemical stability, and impedance matching, titanium nitride exhibits resonance in the visible range as was reported by Qu *et al*.^36^. Ean *et al*.^37^ synthesised an inter-metallic titanium nitride compound that could be used as a diffusion barrier for electronic devices and decorative coating; however, the authors only investigated its negative permittivity in the radio frequency range. Moreover, several carbonaceous materials were tested in the radio frequency range, and their negative permittivity characteristics were compared with those of metallic composites (the former included carbon nanotubes, which possessed extraordinary electrical thermal properties, high mechanical strength, and controllable microstructures). For example, Qu *et al*.^38^ designed carbon nanotubes, whose electromagnetic properties were investigated in the radio frequency range. While all the studies reported in the literature were performed in the radio frequency range, the flexible materials synthesized in this work could be utilised at the microwave frequencies. Moreover, the use of Ni in a composite structure increases its corrosion resistance, toughness, strengths at high and low temperatures, and range of special magnetic and electronic properties.

**Table 3:** Performance analysis for the substrates Ni_36_ and Ni_42_.

References	Substrate material	Substrate type	Negative refractive index bandwidth	Applicable band	Metamaterial type
Islam *et al*.^29^	FR4	Solid	700 MHz	S, C, X, Ku	DNG
Hasan *et al*.^30^	FR4	Solid	2.17 GHz	X, Ku	DNG
Liu *et al*.^31^	FR4	Solid	1 GHz	C, X	DNG
Ziolkowski *et al*.^32^	FR4	Solid	900 MHz	X, Ku	DNG
Joshi *et al*.^33^	Polyester	Flexible	350 MHz	C	SNG
Proposed substrate unit cell	Nickel Aluminate (NiAl_2_O_4_)	Flexible	5.41 GHz (for Ni_36_) 3.30 GHz (for Ni_42_)	S, C, X	DNG

## Conclusions

New flexible NiAl_2_O_4_-based metamaterial substrates with two different compositions and average crystallite sizes of 13–16 nm are synthesised by the sol-gel method, and their dielectric characteristics related to the electromagnetic properties are investigated. The fabricated Ni_36_ and Ni_42_ substrates exhibit high dielectric permittivities of 4.94 to 4.97 and loss tangents of 0.01 and 0.007, respectively. Moreover, the produced flexible substrate materials behave like double negative metamaterials at frequencies of 7.42 GHz and 11.84 GHz for Ni_36_ and 7.43 GHz and 11.85 GHz for Ni_42_, which confirm their applicability in the microwave frequency range, including its S-, C-, and X-bands. In addition, a good match between the simulated and measured values is observed. From the results of this study, it can be concluded that the synthesised materials can be used as flexible metamaterials for electronic and magnetic devices operated in the microwave regime because of their high magnetic permeabilities and low magnetic losses.
